# SVM-Based Spectrum Mobility Prediction Scheme in Mobile Cognitive Radio Networks

**DOI:** 10.1155/2014/395212

**Published:** 2014-03-30

**Authors:** Yao Wang, Zhongzhao Zhang, Lin Ma, Jiamei Chen

**Affiliations:** ^1^Communication Research Center, Harbin Institute of Technology, Harbin 150080, China; ^2^Communication Department, Shenyang Artillery Academy, Shenyang 110867, China

## Abstract

Spectrum mobility as an essential issue has not been fully investigated in mobile cognitive radio networks (CRNs). In this paper, a novel support vector machine based spectrum mobility prediction (SVM-SMP) scheme is presented considering time-varying and space-varying characteristics simultaneously in mobile CRNs. The mobility of cognitive users (CUs) and the working activities of primary users (PUs) are analyzed in theory. And a joint feature vector extraction (JFVE) method is proposed based on the theoretical analysis. Then spectrum mobility prediction is executed through the classification of SVM with a fast convergence speed. Numerical results validate that SVM-SMP gains better short-time prediction accuracy rate and miss prediction rate performance than the two algorithms just depending on the location and speed information. Additionally, a rational parameter design can remedy the prediction performance degradation caused by high speed SUs with strong randomness movements.

## 1. Introduction

Cognitive radio (CR) as a solution for the next generation wireless networks brings new hope to address the wireless spectrum inefficiency problem which has attracted a great deal of attention in recent years [1–4]. In general, CR paradigms are classified in three types: interweave, underlay, and overlay. In interweave or opportunistic spectrum access (OSA) model [5, 6], CUs can use the licensed spectrums opportunistically when the spectrums are detected idle by spectrum sensing. It is very sensitive to PU traffic pattern and it relies on the detection error for the models [6, 7]. Thus, it is essential to investigate the spectrum mobility which is the foundation of resource allocation and network construction.

In a CRN, the spectrum mobility for CUs includes two aspects: spectrum mobility in the time domain and spectrum mobility in the space domain [8]. The time-varying and space-varying characteristics of the spectrum mobility lead to the problem that it is hard to access the licensed spectrums for CUs in a real network. Time-varying characteristic is because of the random variations of PUs' arrivals and departures. Thus, some related literatures have focused on the impact of PUs' activity on CRNs [9–11]. In [12], a selective opportunistic spectrum access scheme is proposed with the aid of PUs' traffic prediction techniques. The scheme can estimate the probability of a channel being idle and choose the best order of spectrum sensing to maximize spectrum efficiency. The definition of channel availability vector is introduced to characterize the state information of licensed channels [13]. And a prediction-based sensing approach is presented to maximize system throughput which reduces the sensing time. In [14], a forecast scheme of call arrival rate and call holding time for PUs is proposed. CUs can reduce the frequency hopping rate through the traffic pattern prediction of PUs.

In the space domain, the movement of CUs directly results in the changing of the spectrum availability. Nevertheless, the movement of CUs, as one of the most important factors in wireless communication systems, is not adequately discussed for CRNs in existing works. A mobility model describing airborne nodes is proposed in [15]. And a stability-capacity-adaptive routing scheme is proposed to achieve high throughput and small transmission time based on the model. In [16], an optimal power control algorithm in mobile CR ad hoc networks is proposed. Without causing harmful interference to PUs, the network achieves maximized throughput based on the algorithm in the legacy network. In [17], a cluster-based routing protocol which can increase throughput and reduce data delivery latency is presented to mend the route in mobile CRNs. In [18], a general scheduling framework with the mobility information is conducted to solve maximum throughput channel scheduling problem for mobile CRNs. And two polynomial time optimal algorithms are proposed and evaluated by using the mobility trace obtained from a real public transportation system.

However, few of existing works investigate the following two issues: (1) considering time domain and space domain characteristics of spectrum mobility together and (2) considering the prediction of spectrum mobility. In practice, a CRN should be forward looking rather than reactive [19]. And a prediction-based CRN can not only improve system performance but also minimize interference to PUs [20–22], because spectrum detecting may take a long time or delay. In [23], a neural network based channel status predictor using multilayer perceptron is proposed. The system spectrum utilization is improved and the sensing energy is saved greatly by predicting the idle channels. In [24], a channel handoff scheme based on SVM is presented to reduce the handoff time. The channel handoff caused by the random movement of PUs and CUs is considered in the prediction design. In [25], a binary time series approach is used to predict the future occupancy of neighboring channels. This approach performs very well for deterministic occupancy even without updating data.

The key contributions of this paper are as follows: (1) we first take the two issues discussed above into account at the same time. And an effective joint feature vector extraction scheme is originally designed through the theoretical analysis on joint information of CUs' mobility and PUs' working activities. (2) Based on the extracted joint feature vector, a novel SVM-based spectrum mobility prediction scheme considering the time-space domain of spectrum mobility together is proposed for mobile CRNs in order to ameliorate the traditional prediction methods only utilizing the location and speed information directly. (3) Finally, simulations are conducted to confirm the effectiveness of the proposed prediction mechanism. The new prediction mechanism achieves higher short-time prediction performance than the conventional algorithms with little training nodes, which is vital in CRNs.

The rest of the paper is organized as follows. The system model is described in Section 2 and the spectrum availability of SUs is discussed in Section 3. In Section 4, a spectrum mobility prediction scheme is proposed based on SVM. The simulation results are shown along with a discussion in Section 5. At last, Section 6 concludes the paper.

## 2. System Model

### 2.1. Mobile CRN System Model

**Figure 1:**
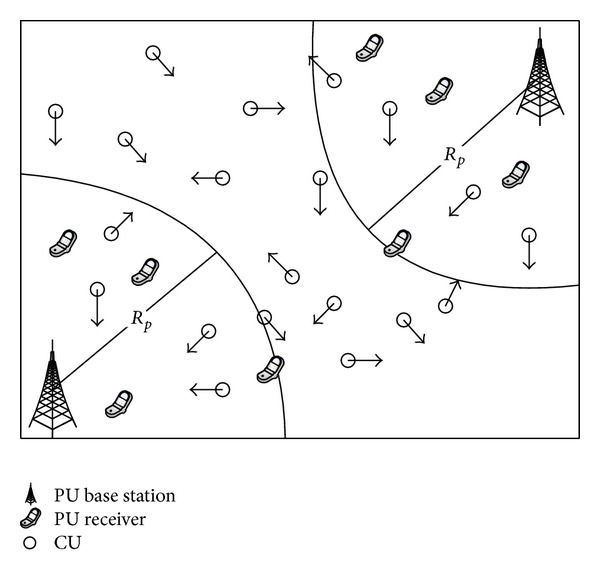
System model of a mobile CRN.

In this paper, we consider a mobile CRN scenario where *N*_*c*_ CUs coexist with *N*_*p*_ PUs illustrated in Figure 1. Assume that each PU_*p*_ (*p* = 1 : *N*_*p*_) has a licensed access to a spectrum *c*_*p*_ with a coverage radius *R*_*p*_. Thus, the number of PUs is equal to the number of spectrums in the network. Each CU_*c*_ (*c* = 1 : *N*_*c*_), with an interference radius *r*, can exploit locally unused licensed spectrum opportunistically without causing any interference to the corresponding PU_*p*_. Suppose that the CRN assigns spectrums periodically with an allocation interval time *T*_*c*_ which is the interval time between two times of spectrum allocation. We also assume spectrum sensing is ideal in this paper.

Figure 1 gives out an instantaneous snapshot of a mobile CRN deployment with 20 mobile CUs. Two PUs are located in the area. The activity of each PU_*p*_(*p* = 2 in Figure 1) is characterized as an on/off (busy/idle) model. The busy time and idle time of PU_*p*_can be modeled by the exponential distribution with means *α*_*p*_ and *β*_*p*_, respectively [26–28]. The probability density function (PDF) can be written, respectively, as

(1) fON(t,αp)=1αpe−t/αp, t≥0,fOFF(t,βp)=1βpe−t/βp, t≥0.

In this paper, a random mobility model which characterizes the movement of CUs in a two-dimensional space is considered [29]. The movement of each CU_*c*_ consists of a sequence of random length intervals called mobility epochs during which CU_*c*_ moves at a constant speed in a constant direction. And the mobility epoch lengths *T*_*e*_ are independently exponentially distributed with mean 1/*λ*_*e*_. The probability distribution function can be expressed as

(2) Me(x)=P(Te≤x)=1−e−λex.

**Figure 2:**
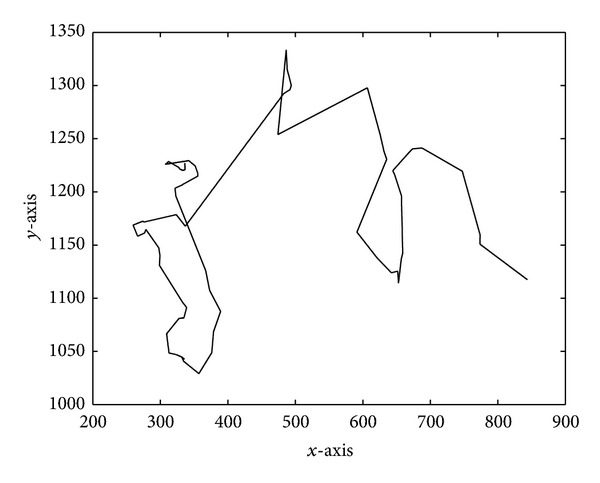
A mobility trajectory example for one given CU_*c*_.

During each epoch, the mobile direction of CU_*c*_ is uniformly distributed over [0,2*π*) and the speed of CU_*c*_ is uniformly distributed over [0, *v*_max⁡_]. We assume mobility is uncorrelated among all the CUs in a network. And it is reasonable to assume that epoch length, speed, and direction are uncorrelated in the model.  Figure 2 shows a mobility trajectory of one given CU_*c*_ as an example.

**Definition 1**

Given a licensed spectrum *c*_*p*_ and an instantaneous time *t*, the instantaneous spectrum availability ISA_*p*_^*c*^(*t*) for one CU_*c*_ can be defined as

(3) ISApc(t)={−1Dp,c(t)<Rp+rc∩(αp(t)=−1)1Dp,c(t)<Rp+rc∩(αp(t)=1)1Dp,c(t)≥Rp+rc.

ISA_*p*_^*c*^(*t*) = 1 means that licensed spectrum *c*_*p*_ is instantaneously available at *t* for CU_*c*_ and ISA_*p*_^*c*^(*t*) = −1 means that licensed spectrum *c*_*p*_ is not instantaneous available at *t* for CU_*c*_, where *D*_*p*,__*c*_(*t*) represents the Euclidean distance between PU_*p*_ and CU_*c*_ at *t*. *α*_*p*_(*t*) represents the time-varying working activity of PU_*p*_, denoted as

(4) αp(t)={1PUp    is  idle  at  t−1PUp    is  busy  at  t.

**Definition 2**

Given a licensed spectrum *c*_*p*_ and a continuous period *T*, the continuous spectrum availability CSA_*p*_^*c*^(*T*) for one CU_*c*_ can be defined as

(5) CSApc(T)={(c,p) ∣ ISApc(t)=1  from  t0  to  t0+T},

where *t*_0_ is a given reference time. CSA_*p*_^*c*^(*T*) = 1 means that spectrum *c*_*p*_ is available to CU_*c*_ not only at *t*_0_ but also at any time between *t*_0_ and *t*_0_ + *T*. CSA_*p*_^*c*^(*T*) = 1 means that spectrum *c*_*p*_ is not available to CU_*c*_ at some time between *t*_0_ and *t*_0_ + *T*. In practice, *T* could be a slot or some slots, during which CU_*c*_ can achieve activation and access to the network. Apparently, we focus more on CSA_*p*_^*c*^(*T*) because a continuous time period (or a short-time duration) rather than an instantaneous time is more meaningful for OSA or resource allocation in CRN.

Our objective is to give out the spectrum mobility prediction for all the CUs based on the joint theoretical analysis of CUs' mobility and PUs' working activities under the mobile model. Obviously, it is a complicated nonlinear problem which cannot be solved by normal algorithm. In this paper, a new prediction scheme is presented to solve this problem by SVM.

### 2.2. Support Vector Machine

As a highly competitive learning method, SVM is gaining popularity in many fields based on the statistical learning theory [30–32]. SVM adopts structural risk minimization principle which has been shown superior to empirical risk minimization principle used by traditional neural networks [24]. Moreover, the generalization ability of SVM is strong [33]. SVM is initially used to solve the classification problem. Assume there is *l* training sample data denoted as

(6) $$\begin{array}{c} D={\left\{\left(x_{i},y_{i}\right)\;|\;x_{i}\in R^{d}\right\}}_{i=1}^{l}, \end{array}$$

where **x**_*i*_ is an input vector containing multiple features. *y*_*i*_ ∈ {−1, +1} is a class indicator. *d* is the dimension of sample data. Optimal hyper plane is constructed as

(7) $$\begin{array}{c} w\cdot x_{i}+b=0, \end{array}$$

where **w** are weights and *b* is offset argument. The samples on *H*1 and *H*2 are support vectors. The according equations are **w** · **x**_*i*_ + *b* = +1 and **w** · **x**_*i*_ + *b* = −1, respectively. So, the classification margin is 2/||**ω**||. For our nonlinear problem, the representation of the sample data has to be changed from the original input space to a higher dimensional space which is referred to as the feature space. This quadratic programming (QP) problem can be expressed as

(8) min⁡ Φ(w,ξ)=12||w||2+C∑i=1lξi,s.t. yi[(w·xi)+b]≥1−ξi, i=1:l.

*ξ*_*i*_ is relaxation factor and *C* is cost parameter which is a given value. A Lagrange function is constructed to solve the above constrained optimization problem (8) as follows:

(9) Q(w,b,α,β,ξ)=12||w||2+C∑i=1lξi −∑i=1lαi[ci(xi·w+b)−1+ξi]−∑i=1lβiξi,

where *α*_*i*_ and *β*_*i*_ are Lagrange multipliers. In order to get the solution of the original problem, we calculate the partial derivative for different variables as

(10) ∂Q(w,b,α,β,ξ)∂w=w−∑i=1lαicixi=0,∂Q(w,b,α,β,ξ)∂b=∑i=1lαici=0,∂Q(w,b,α,β,ξ)∂ξ=C−αi−βi=0.

Based on (10), the original optimization problem shown in (9) is transformed to a dual optimization problem as

(11) max⁡ J(α)=∑i=1lαi−12∑i=1,j=1lαiαjcicjK(xi,xj),s.t. 0≤αi≤C,∑i=1lαici′=0, i=1,2,…,l.

*K*(**x**_*i*_, **x**_*j*_) is a chosen kernel function which will be discussed later in the prediction scheme section. Thus, we can obtain the optimization w*

(12) w∗=∑i=1lαi∗ci′xi,

where *α*_i_* can be solved by (11). And *b* which does not appear in the dual problem can be calculated through the original constraint. Consider

(13) $$\begin{array}{c} b^{*}=\frac{1}{2}\left[\max_{c_{i}=-1}\left(\langle w^{*}\cdot x_{i}\rangle \right)+\min_{c_{i}=+1}\left(\langle w^{*}\cdot x_{i}\rangle \right)\right]. \end{array}$$

Therefore, the final prediction output expression can be written as

(14) $$\begin{array}{c} D\left(l^{\prime }\right)=\mathrm{sign}\left(\overset{l}{\underset{i=1}{\sum }}\alpha_{i}c_{i}K\left(x_{i},x_{j}\right)+b\right). \end{array}$$

## 3. Joint Feature Vector Extraction

The common idea is utilizing SVM via domain information such as location and speed directly. However, it does not make good use of the CUs' mobility characteristic and PUs' working activities information. The traditional methods, thus, result in low prediction accuracy performance, which will be discussed in the simulation part.

In this section, CSA_*p*_^*c*^(*T*) as joint feature vector is extracted for SVM through theoretical deduction. Obviously, CSA_*p*_^*c*^(*T*) is related to a period *T*. In this paper, we focus mainly on spectrum allocation interval time *T*_*c*_ which is meaningful for a real CRN. Two situations need to be investigated in order to derive CSA_*p*_^*c*^(*T*_*c*_): (1) CU_*c*_ is in the coverage area of PU_*p*_ at *t*_0_ and (2) CU_*c*_ is not in the coverage area of PU_*p*_ at *t*_0_, respectively.

For the first situation, let CU_*c*_ predict a period *T*_*p*_ during which CU_*c*_ can use *c*_*p*_ continuously. And our idea is to derive CSA_*p*_^*c*^(*T*_*c*_) through computing CSA_*p*_^*c*^(*T*_*p*_). In fact, CSA_*p*_^*c*^(*T*_*p*_) includes two main situations: (1) *C*_1_(*T*_*p*_) representing the situation that CU_*c*_ does not move into PU_*p*_′ coverage scope between *t*_0_ and *t*_0_ + *T*_*p*_ and (2) *C*_2_(*T*_*p*_) representing the situation that CU_*c*_ moves into PU_*p*_′ coverage scope at *t*_0_ + *T*_*s*_  (0 ≤ *T*_*s*_ ≤ *T*_*p*_) while the activity of PU_*p*_ is inactive between *t*_0_ + *T*_*s*_ and *t*_0_ + *T*_*p*_. We believe *C*_1_(*T*_*p*_) and *C*_2_(*T*_*p*_) dominate the main situations although other complicated situations as small probability events also exist.

*C*_1_(*T*_*p*_) also contains two parts: (1) *P*_out1_ representing the situation that the velocity of CU_*c*_ does not change from *t*_0_ to *t*_0_ + *T*_*p*_ and (2) *P*_out2_ representing the other situations. Consider

(15) $$\begin{array}{c} C_{1}\left(T_{p}\right)=P_{\text{out}1}+P_{\text{out}2}. \end{array}$$

From (2), *P*_out1_ can be easily obtained as

(16) $$\begin{array}{c} P_{\text{out}1}=1-M_{e}\left(T_{p}\right)=e^{-\lambda_{e}T_{p}}. \end{array}$$

It is difficult to get the accurate value of *P*_out2_ because we cannot know the velocity change information (the change in time, speed, and direction) at any time for CU_*c*_. However, the approximate value *E*(*P*_out2_) by estimating *P*_out2_ can be derived [34]. When *T*_*p*_ < *T*_*c*_, CU_*c*_ has to change its movement speed and direction (or any of them) before *t*_0_ + *T*_*p*_, which makes CU_*c*_ away from PU_*p*_. Therefore, CSA_*p*_^*c*^(*T*_*c*_) can be obtained as

(17) CSApc(Tc)≈E(Pout2)=1λeTp+εa +e−λeTp(12pawayλeTp−1λeTp−εa−1),

where *p*_away_ denotes the probability that CU_*c*_ moves away from PU_*p*_ after the first velocity (speed and direction) change. In practice, *ε*_*a*_ ≥ 0 represents other situations (small probability events except the situations discussed above). For example, CU_*c*_ changes its speed three times while CU_*c*_ still does not move into PU_*p*_'s coverage scope from *t*_0_ to *t*_0_ + *T*_*c*_. *ε*_*a*_ is used to balance the equation and we will discuss it later.

When *T*_*p*_ ≥ *T*_*c*_, CSA_*p*_^*c*^(*T*_*c*_) for CU_*c*_ can be obtained as

(18) CSApc(Tc)≈C1(Tc)+E(Pout2)=1λeTc+εa+e−λeTc(12pawayλeTc−1λeTc−εa).

For the second situation, the movement of CU_*c*_ and the working state of PU_*p*_ should be investigated simultaneously. Different from the first situation, we believe that the prediction of the idle state for PU_*p*_ is more essential due to the original position of CU_*c*_. Similar to the first situation, let CU_*c*_ predict a continuous period *T*_*p*_^out^ that CU_*c*_ will not move out of PU_*p*_'s coverage boundary from *t*_0_ to *t*_0_ + *T*_*p*_^out^. It is noted that *T*_*p*_^out^ is different from the *T*_*p*_ mentioned above.

When *T*_*p*_^out^ < *T*_*c*_, CSA_*p*_^*c*^(*T*_*c*_) for CU_*c*_ can be obtained as

(19) $$\begin{array}{c} {\text{CSA}}_{p}^{c}\left(T_{c}\right)=p_{\text{in}}\cdot \overset{t_{0}+T_{p}^{\text{out}}}{\underset{t_{0}}{\int }}f_{\text{OFF}}\left(t,\beta_{p}\right)dt+\varepsilon_{\text{in}}. \end{array}$$

Similar to *ε*_*a*_ above, *ε*_in_ is used to denote all the other small probability events. ∫_*t*_0__^*t*_0_+*T*_*p*_^out^^ *f*_OFF_(*t*)*dt* represents the idle probability of *c*_*p*_ between *t*_0_ and *t*_0_ + *T*_*p*_^out^. *p*_in_ represents the probability that CU_*c*_ moves out of PU_*p*_′ coverage boundary before *t*_0_ + *T*_*p*_. *p*_in_ consists of two parts. *P*_in1_ denotes the situation that the velocity of CU_*c*_ remains unchanged from *t*_0_ to *t*_0_ + *T*_*p*_. And *P*_in2_ denotes the other situations. *P*_in_ can be easily obtained as

(20) $$\begin{array}{c} P_{\text{in}}=\frac{1}{\lambda_{e}T_{p}^{\text{out}}}+\varepsilon_{b}+e^{-\lambda_{e}T_{p}}\left(\frac{1}{2}p_{b}\lambda_{e}T_{p}^{\text{out}}-\frac{1}{\lambda_{e}T_{p}^{\text{out}}}-\varepsilon_{b}\right), \end{array}$$

where *ε*_*b*_ ≥ 0 tries to represent all the other situations. *p*_*b*_ represents the probability that CU_*c*_ moves away from PU_*p*_ after the first change in velocity. According to (1), (19), and (20), CSA_*p*_^*c*^(*T*_*c*_) for CU_*c*_ can be obtained as

(21) CSApc(Tc)=(1λeTpout+εb+e−λeTpout(12pbλeTpout−1λeTpout−εb)) ×(e−μpt0−e−1/βp·(t0+Tpout))+εin.

When *T*_*p*_^out^ ≥ *T*_*c*_, CSA_*p*_^*c*^(*T*_*c*_) is mainly determined by PU_*p*_'s working activity. Thus, CSA_*p*_^*c*^(*T*_*c*_) for CU_*c*_ can be obtained as

(22) CSApc(Tc)=∫t0t0+TcfOFF(t,βp)dt+εc=e−1/βp·t0−e−1/βp·(t0+Tc)+εc.

*ε*_*c*_ denotes all the other spectrum availability situations. Thus, we obtain joint feature vector sets **S**_*i*_^*c*^ = {CSA_*p*_^*c*^(*T*_*c*_)_*i*_}_*i*=1_^*l*^ according to different situations based on (17), (18), (21), and (22). Moreover, we can get the ultimate prediction expression according to (14)

(23) D(l′)=sign⁡(∑i=1mαici′K(Sic,xj)+b),s.t. Sic={CSApc(Tc)i}i=1l.

## 4. Spectrum Mobility Prediction Scheme

In this section, a new SVM-based spectrum mobility prediction scheme is proposed based on the analysis and deduction above. The main steps of the proposed prediction scheme are as follows.

**Step 1 (CRN initialization)**

Initialize the original locations of PUs and CUs randomly in the two-dimensional deployment area. The coordinates of PUs are not changed once generated. And PUs' initial working states are stochastic. Initialize the original speed, direction, and epoch lengths for each CU_*c*_. Assume the maximum velocity for CUs is *v*_max⁡_. The beginning time of the system is set to *t*_0_. The parameters C, *ξ*_*i*_ of SVM are initialized. Simulations are based on many times to make sure of the accuracy of the result. In addition, set *T*_*c*_, *α*_*p*_, *β*_*p*_, *ε*_away_, *ε*_*a*_, *ε*_*b*_, *ε*_*c*_, *p*_away_, *p*_*b*_ *λ*_*e*_, *R*_*p*_, and *r*_*c*_.

**Step 2 (operate the mobile CRN model)**

PUs' working states obey an exponential on/off model. The PDF satisfies (1). And the mobility of CUs follows the mobile model mentioned in Section 2.1. The mobility epoch lengths are independently exponentially distributed with mean 1/*λ*_*e*_. It is noted that wrap-around technique is adopted during simulation in order to make the total number of SUs unchanged in the simulation area.

**Step 3 (calculate the joint feature vectors S_*i*_^*c*^ for SVM)**

Here, there are three situations to be investigated. Firstly, CU_*c*_ does not move into the coverage of PU_*p*_ at *t*_0_ granted that CU_*c*_ moves with *v*_max⁡_ towards PU_*p*_. Secondly, CU_*c*_ is out of the coverage of PU_*p*_ at *t*_0_, but CU_*c*_ may move into the coverage of PU_*p*_ between *t*_0_ and *t*_0_ + *T*_*c*_. Finally, CU_*c*_ is in the coverage of PU_*p*_ at *t*_0_. The detail calculation steps for **S**_*i*_^*c*^ are described in Algorithm 1.

**Step 4 (execute prediction by SVM)**

Firstly, a SVM prediction model is generated according to the history input vectors **S**_*i*_^*c*^ from Step 3. Secondly, put the data to be predicted into the generated SVM model. Then, compute the prediction results and record the results. Here, we adopt the RBF kernel as mapping function for SVM in simulation. Because the RBF kernel function tends to obtain more robust results than other kernels and can reduce numerical difficulties, the RBF kernel function can be defined as

(24) $$\begin{array}{c} K\left(x,y\right)=\exp\left(-\gamma {||x-y||}^{2}\right),\;\gamma >0. \end{array}$$

**Step 5 (the system resets)**

Execute Steps 1 to 4 until simulation numbers are satisfied for testing. Then, the operation stops. Compute the prediction performance: the prediction accuracy rate *p*_accuracy_ and the miss detection probability *p*_miss_. *p*_accuracy_ is defined as

(25) $$\begin{array}{c} p_{\text{accuracy}}=\frac{\left|\left\{i\;|\;y_{i}^{\prime }\cdot f\left(x_{i}^{\prime }\right)>0\right\}\right|}{l^{\prime }}\times 100\%, \end{array}$$

where {**x**_*i*_′}_1_^*l*′^ are testing data which are to be predicted. And *y*_*i*_′ ∈ {−1, +1} are true labels for testing data. *l*′ is the total number of testing data. *f*(**x**_*i*_′) ∈ {−1, +1} are the predicted decision values. Here, {−1, +1} represents the busy/idle working activity for a given PU. |·| represents the element numbers for a given set. Actually, *p*_accuracy_ reflects the accuracy degree of spectrum prediction mechanism. The higher the *p*_accuracy_ is, the better the prediction effect is.

In addition, the miss prediction rate *p*_miss_ (the rate that the spectrum is predicted to be idle while it is actually busy) is investigated for the proposed prediction mechanism. Because *p*_miss_ can reflect the actual interference to the PUs to some extent. The smaller the *p*_miss_ is, the better the prediction mechanism is. *p*_miss_ can be defined as

(26) $$\begin{array}{c} p_{\text{miss}}=\frac{\left|\left\{i\;|\;y_{i}^{\prime }\cdot f\left(x_{i}^{\prime }\right)<0,f\left(x_{i}^{\prime }\right)=1\right\}\right|}{l^{\prime }}\times 100\%. \end{array}$$

Note that *p*_accuracy_ + *p*_miss_ ≤ 100%.

## 5. **Simulation Results and Analysis**

In this section, experimental results of the prediction performances for our proposed scheme are investigated. Simulation parameters are shown in Table 1. We compare the proposed prediction mechanism with the traditional prediction schemes (SVM with initial location coordinates of CUs and SVM with initial location coordinates and speed of CUs) to evaluate the prediction performances under different parameters.

Note that we assume *ε*_*a*_ = *ε*_*b*_ = *ε*_*c*_ = *ε*_in_ = *ε*_away_ during simulation for simplicity, because they are very small positive values that are set to balance the according equations. In addition, the total testing number is set to 1000 in order to avoid randomness during simulation.

**Figure 3:**
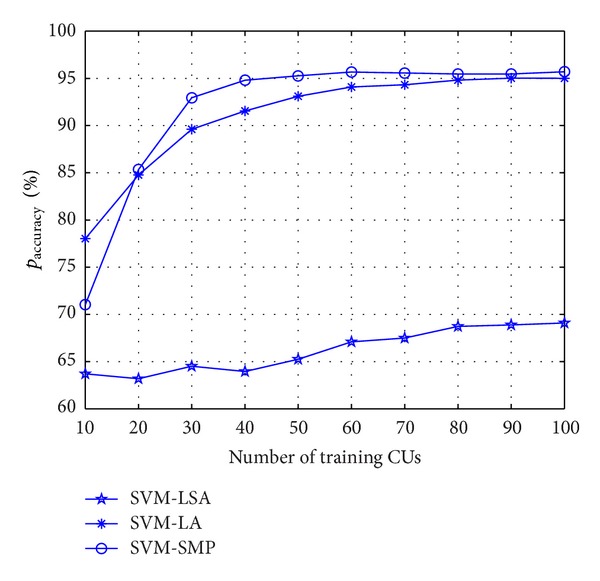
Prediction accuracy rate *p*_accuracy_ for CUs versus training node number.

Figure 3 shows *p*_accuracy_ among different algorithms versus training node number. The proposed SVM-SMP converges at about 40 training data faster than SVM-location algorithm (SVM-LA) with a convergent result at about 100 training data. *p*_accuracy_ of SVM-location-speed algorithm (SVM-LSA) is much worse than the other comparison algorithms which shows that the initial speed parameters have a bad effect on the prediction performance. It is caused by the time-varying characteristic of CUs' velocity (speed or direction).

**Figure 4:**
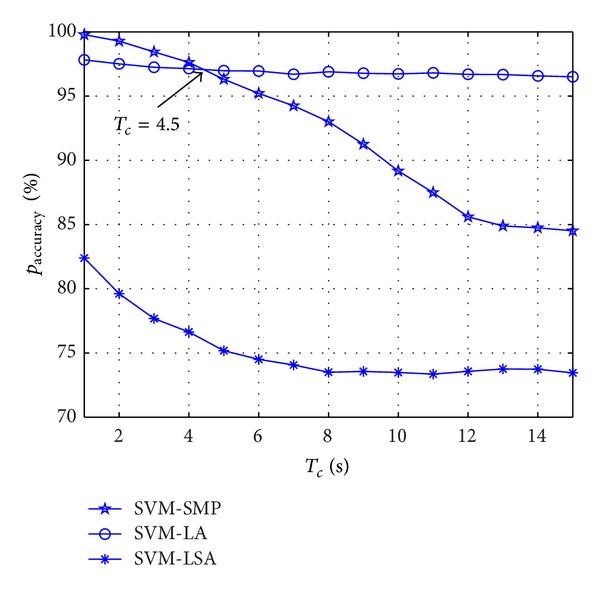
Prediction accuracy rate *p*_accuracy_ for CUs versus *T*_*c*_.

As shown in Figure 4, *p*_accuracy_ decreases with the increasing of *T*_*c*_ for the three different algorithms. *p*_accuracy_ of the proposed SVM-SMP is better than SVM-LA when *T*_*c*_ is relatively small (1 ≤ *T*_*c*_ ≤ 4.5). However, *p*_accuracy_ of SVM-SMP is worse than SVM-LA when *T*_*c*_ is big enough (*T*_*c*_ > 4.5). It is because the proposed prediction scheme is based on the short-time prediction idea according to the feature vector extraction analysis in (17), (18), (21), and (22). Moreover, wrap-around technique makes CUs stay at their original positions with big probability at the simulation boundary. Thus, SVM-LA decreases not that fast with the increasing of *T*_*c*_. And the SVM-SMP works well when the prediction time is not long and vice versa. Note that the short-time prediction performance is mainly focused on in this paper because CR itself should achieve communication in a very short time.

**Figure 5:**
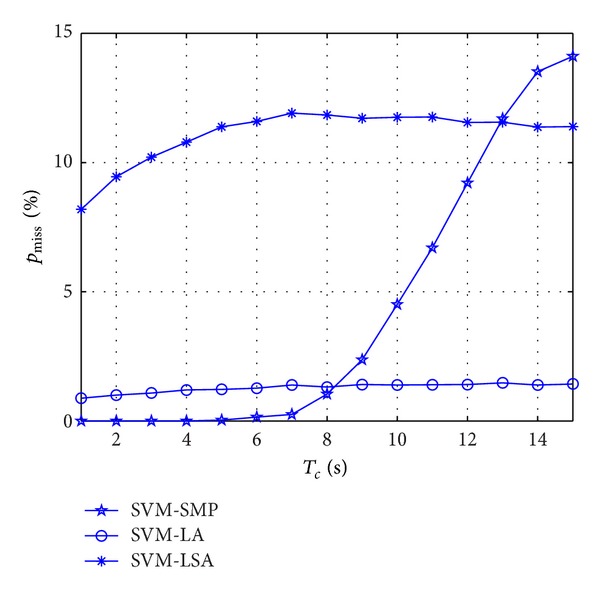
Miss prediction rate *p*_miss_ for CUs versus *T*_*c*_.

As illustrated in Figure 5, *p*_miss_ is studied versus *T*_*c*_ for different algorithms. *p*_miss_ of SVM-SMP is nearly equal to 0 when *T*_*c*_ is small (1 ≤ *T*_*c*_ ≤ 5), which is better than SVM-LA. However, *p*_miss_ of SVM-SMP increases very fast when *T*_*c*_ is big enough. Figure 5 shows the good short-time miss prediction rate characteristics of the new algorithm which is very essential to CR.

**Figure 6:**
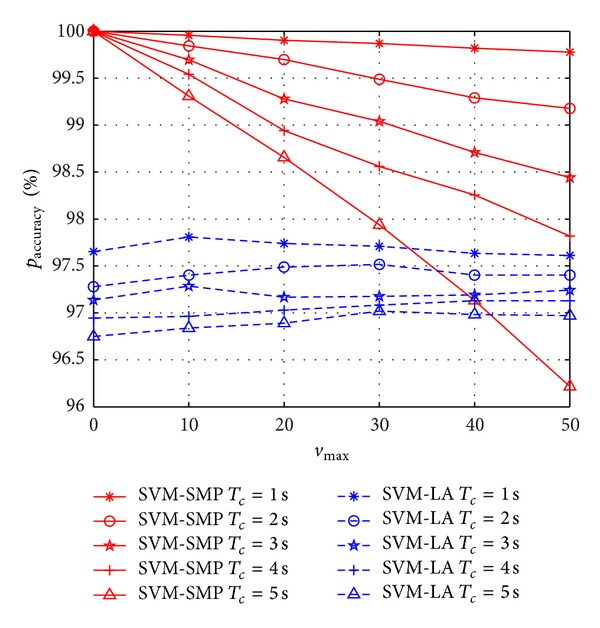
Prediction accuracy rate *p*_accuracy_ for CUs versus *v*_max⁡_.

**Figure 7:**
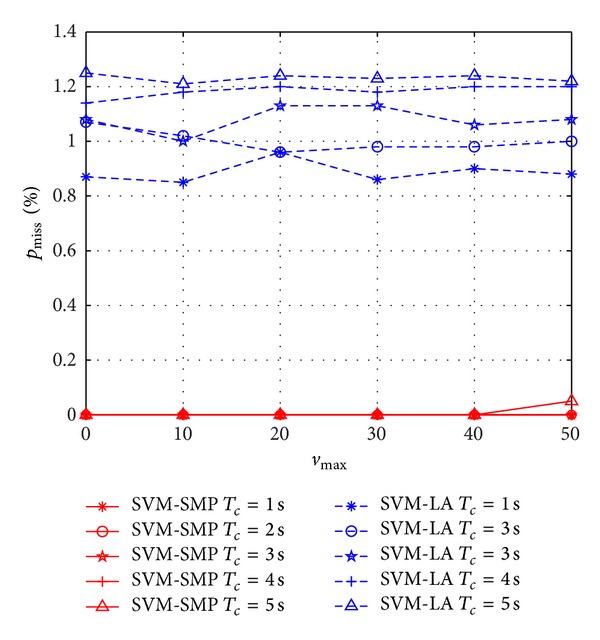
Miss prediction rate *p*_miss_ for CUs versus *v*_max⁡_.

In Figures 6 and 7, *p*_accuracy_ and *p*_miss_ versus *v*_max⁡_ are investigated between SVM-SMP and SVM-LA. From Figure 6, *p*_accuracy_ of SVM-SMP is better than that of SVM-LA when 1 ≤ *T*_*c*_ ≤ 4 s. However, *p*_accuracy_ of SVM-SMP is worse than that of SVM-LA when *T*_*c*_ = 5 s and *v*_max⁡_ > 42 m/s. It shows that the proposed SVM-SMP lose the advantages when the prediction time and speed are too big simultaneously. As shown in Figure 7, *p*_miss_ of SVM-SMP is less than 0.1%. Comparatively, *p*_miss_ of SVM-LA is approximately 1%. Thus, SVM-SMP shows good *p*_miss_ performance with *v*_max⁡_ changing.

**Figure 8:**
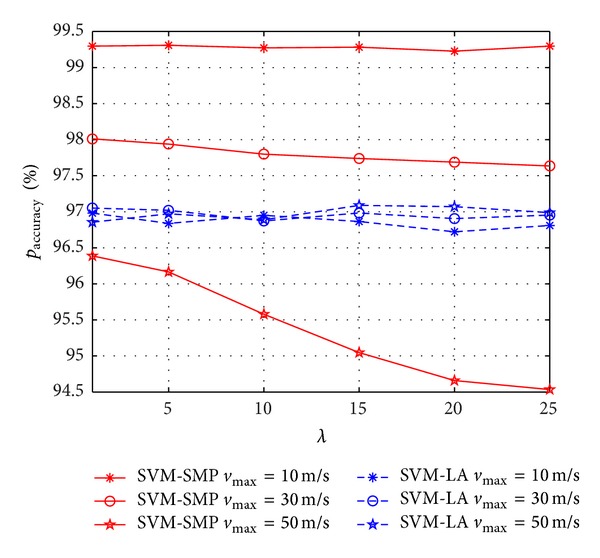
Prediction accuracy rate *p*_accuracy_ for CUs versus *λ*.

**Figure 9:**
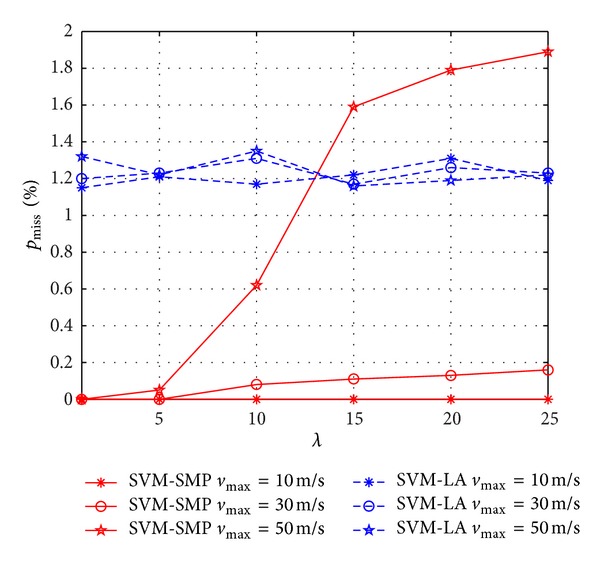
Miss prediction rate *p*_miss_ for CUs versus *λ*.

In Figures 8 and 9, we investigate *p*_accuracy_ and *p*_miss_ versus *λ* between SVM-SMP and SVM-LA. From Figure 8, *p*_accuracy_ of SVM-SMP decreases obviously with the increasing of *λ* when *v*_max⁡_ is big (such as 50 m/s). However, *p*_accuracy_ of SVM-SMP does not change very obviously with the increasing of *λ* when *v*_max⁡_ is small (such as 10 m/s). It is due to the fact that the bigger the *λ* is, the stronger the irregular movements of CUs are. Thus, it is difficult for the prediction when CUs are moving with high speed and strong irregular movements. As shown in Figure 9, *p*_miss_ of SVM-SMP is much better than that of SVM-LA when *v*_max⁡_ is small (such as 10 m/s, 30 m/s). However, *p*_miss_ of SVM-SMP is worse than that of SVM-LA when *v*_max⁡_ = 50 m/s and *λ* > 13, which validates the performance degradation of the prediction performance again when *v*_max⁡_ is relatively big with strong irregular movements. It is because SVM-SMP is based on the assumption of weak irregular movements for CUs.

**Figure 10:**
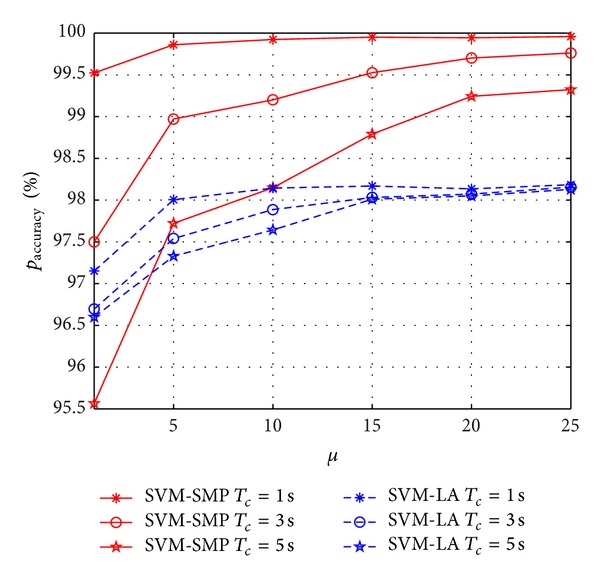
Prediction accuracy rate *p*_accuracy_ for CUs versus *μ*.

**Figure 11:**
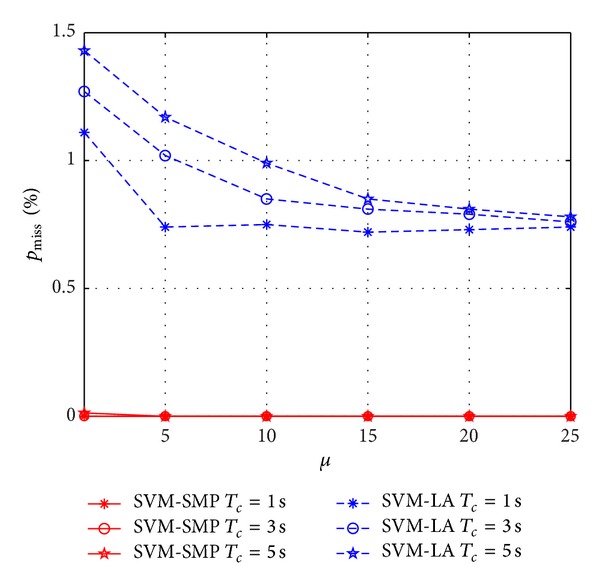
Miss prediction rate *p*_miss_ for CUs versus *μ*.

In Figures 10 and 11, *p*_accuracy_ and *p*_miss_ versus *β*_*p*_ are investigated for SVM-SMP and SVM-LA. In Figure 10, *p*_accuracy_ of SVM-SMP is obviously better than *p*_accuracy_ of SVM-LA when the prediction time is short such as 1 s and 3 s. However, *p*_accuracy_ of SVM-SMP is worse than *p*_accuracy_ of SVM-LA when the prediction duration time is *T*_*c*_ = 5 s and the mean idle time is *β*_*p*_ < 4. In Figure 11, *p*_miss_ of SVM-SMP is nearly equal to 0, which is much better than SVM-LA. In addition, the prediction performance (*p*_accuracy_ and *p*_miss_) does not improve significantly for the two algorithms with the increasing of *β*_*p*_ when *β*_*p*_ is big enough according to Figures 10 and 11.

**Figure 12:**
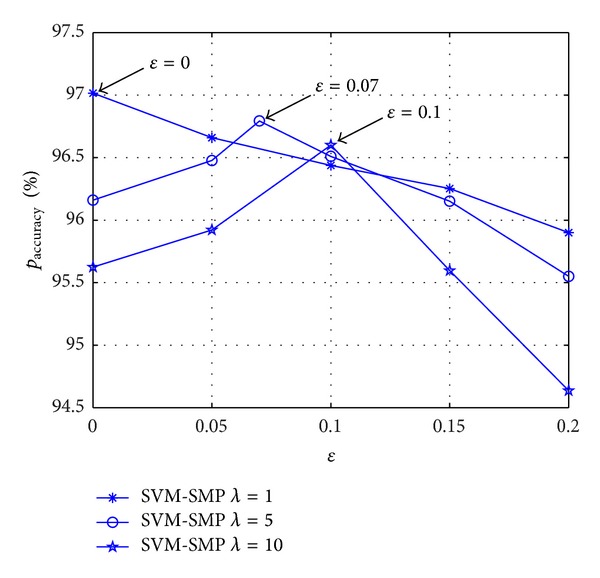
Prediction accuracy rate *p*_accuracy_ for CUs versus *ε*.

Figure 12 shows the impact of *ε* on the prediction accuracy rate *p*_accuracy_ for SVM-SMP. When *λ* = 1, the maximum of *p*_accuracy_ occurs at about *ε* = 0 which is relatively small. With the increasing of *λ*, the maximum value position of *p*_accuracy_ moves to the right. When *λ* = 10, the maximum of *p*_accuracy_ occurs at about *ε* = 0.1. It is because the small probability events happen more times when SUs' randomness movements are strong (*λ* is big). The bigger the *λ* is, the bigger the maximum value position of *p*_accuracy_ occurs for *ε*. Therefore, we can obtain better prediction performance by adjusting *ε* for randomness movements of different strength.

**Figure 13:**
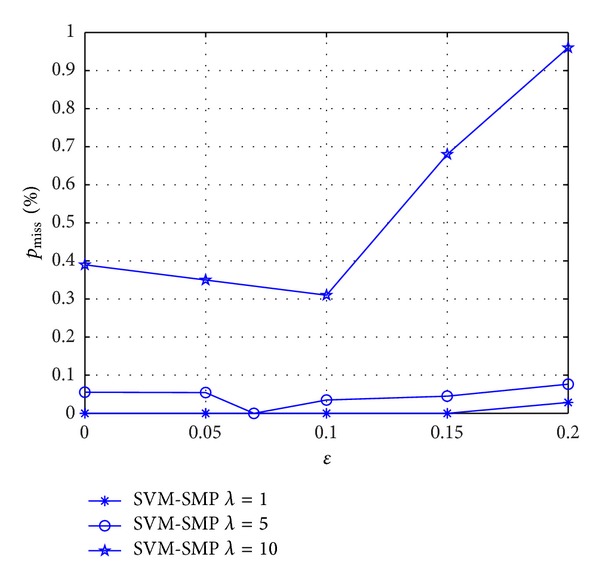
Miss prediction rate *p*_miss_ for CUs versus *ε*.

As shown in Figure 13, miss prediction rate *p*_miss_ is studied versus *ε* for SVM-SMP. When *λ* = 5 and *λ* = 10, the minimum of *p*_miss_ occurs at about *ε* = 0.07 and *ε* = 0.1, respectively. It shows that different optimal *ε* corresponds to SUs' randomness movements of different strength for *p*_miss_. The simulation results further confirm the impact of *ε* on the prediction accuracy rate in Figure 12.

## 6. Conclusions

In this paper, a new spectrum mobility prediction algorithm is proposed in mobile CRNs. SVM theory is adopted to improve the spectrum mobility prediction performance, which takes into account time- and space-varying characteristics together. Moreover, new extracted feature vectors based on the theoretical analysis are input into SVM. Simulation results confirm that the convergence speed of our SVM-SMP is faster than SVM-LA and SVM-LSA. Meanwhile, SVM-SMP shows better short-time prediction performance than SVM-LA and SVM-LSA, which is essential to real mobile CRNs. In addition, the prediction performance degradation caused by SUs' high speed and strong randomness movements can be made up by choosing the proper parameters.

As known to us, how to choose the best parameters (*C* and *ξ*_*i*_) quickly is still an open problem in SVM. We will further analyze the impact of *ε* on prediction performance. It leaves us to investigate in the future.

## Figures and Tables

**Algorithm 1:**
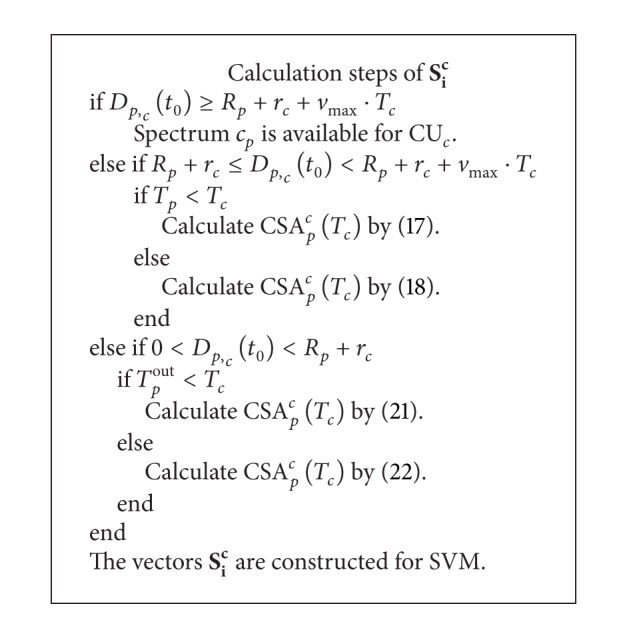
Joint feature vectors extraction for CU_*c*_.

**Table 1:** Simulation parameters.

Parameter	Value
Total simulation number for testing	1000
Simulation area	5000 m × 5000 m
*R*_*p*_	1000 m
*r*_*c*_	500 m
Kernel function	RBF kernel
*C*	10
*ξ*_*i*_	0.01
*T*_*c*_	1 s~15 s
*α*_*p*_	1/3 s
*β*_*p*_	1/3 s
*p*_a_ = *p*_*b*_ = *p*_away_	0.5
*ε*_*a*_ = *ε*_*b*_ = *ε*_*c*_ = *ε*_in_ = *ε*_away_	0~0.2
1/*λ*_*e*_	3 s
CUs' maximum velocity *v*_max⁡_	0 m/s~50 m/s
Total number of PUs	2
Total number of training CUs	20~240
Total number of testing CUs	1000
